# *N*-dimensional measurement-device-independent quantum key distribution with *N* + 1 un-characterized sources: zero quantum-bit-error-rate case

**DOI:** 10.1038/srep30036

**Published:** 2016-07-25

**Authors:** Won-Young Hwang, Hong-Yi Su, Joonwoo Bae

**Affiliations:** 1 Department of Physics Education, Chonnam National University, Gwangju 61186, Republic of Korea; 2 Department of Applied Mathematics, Hanyang University (ERICA), Ansan, Gyeonggi-do, 15588, Republic of Korea

## Abstract

We study *N*-dimensional measurement-device-independent quantum-key-distribution protocol where one checking state is used. Only assuming that the checking state is a superposition of other *N* sources, we show that the protocol is secure in zero quantum-bit-error-rate case, suggesting possibility of the protocol. The method may be applied in other quantum information processing.

Quantum key distribution (QKD)^1^,^2^ enables two remote users, normally called Alice and Bob, to generate key (private random sequence), which is not a possible task classically. QKD is not only a practically important field but also a theoretically appealing one.

After security of QKD for ideal devices was shown^1^,^3^,^4^, problems due to imperfect devices protruded. Although a main problem due to imperfect source was resolved^5^, problem due to imperfect detectors still had remained^6^,^7^. Then no-signaling QKD was discovered^8^,^9^. Remarkably, the no-signaling QKD’s were found to be immune against the imperfect device problems, because security analysis of the protocol is based only on outcomes of detectors. Soon device-independent (DI) QKD’s were found^10^. DI QKD has ideal security but not yet feasible. Measurement-device-independent (MDI) QKD was proposed^11^ and demonstrated^12^,^13^,^14^,^15^ in the background. MDI QKD is secure provided that source is ideal, that is, source is exactly in prescribed quantum states. Later protocols^16^,^17^ with more relaxed condition adapt un-characterized source. The only assumption is that the sources are within 2-dimensional subspace.

It can be expected that MDI QKD can be generalized to *N*-dimensional case. However, security of MDI QKD with un-characterized source relies^16^,^17^ on Shor-Preskill proof^4^. Thus it is not yet clear that *N*-dimensional MDI QKD with un-characterized source works. In this paper, we consider the *N*-dimensional MDI QKD with un-characterized source. The only assumption for security is that the sources are within *N*-dimensional subspace. In the protocol, a single quantum state is enough for checking eavesdropper, normally called Eve. (It is known that a single checking state is enough^18^). We show that the protocol is secure in zero quantum-bit-error-rate (QBER) case. This suggests possibility of *N*-dimensional MDI QKD with un-characterized source.

## Results

For the protocol, each user prepares *N* encoding states. Let the states prepared by Alice and Bob denoted by |*φ*_*m*_〉 and 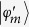, respectively, where *m* = 0, 1, 2, …, *N* − 1. Here nothing is assumed for the encoding states so they are completely un-characterized. Each user also prepares a checking state *which is assumed to be a superposition of the encoding states*. Alice’s and Bob’s checking states are, respectively,


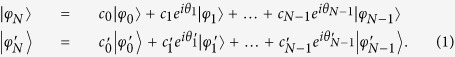


Here *c*_*m*_ and 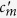 are real numbers with constraints 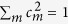 and 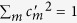, respectively, and *θ*_*m*_ and 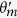 are real. The protocol is as follows.

(1) Alice generates a random number *i* where *i* = 0, 1, 2, …, *N*. She sends a state |*φ*_*i*_〉 to Charlie. Here Charlie can be anyone. So Charlie can be either Eve or users themselves. (2) Bob independently generates a random number *j* where *j* = 0, 1, 2, …, *N*. He sends a state 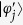 to Charlie. (3) Charlie performs a measurement on set of the states |*φ*_*i*_〉 and 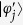. The measurement can be any one which finally gives two outcomes 0 and 1. Charlie announces the outcome. (4) When the outcome is 0, users discard the data. Otherwise, they keep the data. By sacrificing some of the data for public discussion, users estimate, *p*(1|*ij*) ≡ *p*_*ij*_, conditional probability to get outcome 1 for each *i, j*. (5) For each measurement, if both *i* and *j* are less than *N*, the *i* and *j* become raw key. Otherwise, the data are used only for checking purposes. Then users do post-processing to get final key.

Now let us consider Eve’s (Charlie’s) measurement on the states |*φ*_*i*_〉 and 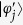. In the most general collective attack, Eve attaches an ancilla |*e*〉 to the states and then applies a unitary operation to them^17^


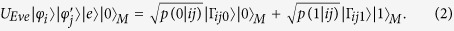


Eve gets the outcome by measuring the quantum state indexed by *M* in basis of |0〉 and |1〉. Now let us consider the attack from Eve’s viewpoint. Clearly she can get no information about key from the data with outcome 0 which are not used by the users. Thus she analyze the states for outcome 1, |Γ_*ij*1_〉’s. For convenience, let us omit 1, |Γ_*ij*1_〉 ≡ |Γ_*ij*_〉. We can see that Eqs (1) and (2) give constraints


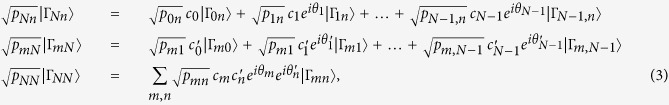


where *n* = 0, 1, …, *N* − 1 and 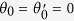. Then Eve’s goal is to maximize *I*_*EA*_ her information about Alice’s or *I*_*EB*_ her information about Bob’s. Eve is constrained only by Eq. (3) and the conditional probabilities *p*_*ij*_’s. Let us consider the case when Eve maximizes *I*_*EA*_. In this case she should discriminate mixed states 
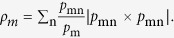
 So the *I*_*EA*_ is bounded by^1^


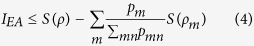


where 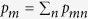 and 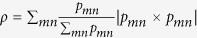. Analogously we also get a bound


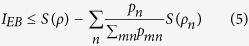


where 
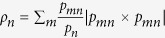
 and 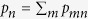. The bounds can be used to obtain key rate^1^.

Now let us consider zero QBER case when *p*_*mn*_ = *δ*_*mn*_(1/*N*), *p*_*mN*_ = *p*_*Nn*_ = 1/*N*^2^, and *p*_*NN*_ = 1/*N*. Here *δ*_*mn*_ is Kronecker delta. This conditional probabilities can be obtained by choosing that |*φ*_*m*_〉 = |*m*〉, 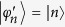, 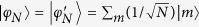, and Eve’s measurement is a one composed of 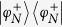 and 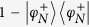 with outcome 1 and 0, respectively. Here |*m*〉’s are mutually orthonormal states and a generalized Bell state 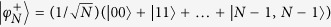. However, here we assume nothing about the states and measurements. Conversely, we show that if the conditional probabilities were obtained anyhow, we can get security and the states should be such. By inserting the conditional probabilities to the first and second of Eq. (3), we get 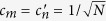. Combining this with the third of Eq. (3), we obtain 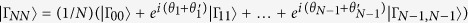. Now by normalization condition (|Γ_*NN*_〉, |Γ_*NN*_〉) = 1, we get


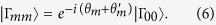


Eq. (6) means that all |Γ_*mm*_〉’s are essentially identical and that righthandside terms in Eqs (4) and (5) are zero, implying the security. Moreover, combining 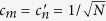, Eq. (1), and normalization condition, we obtain that all |*φ*_*m*_〉’s are orthogonal with one another and the same property holds for 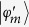
’s. We can also see that the states |*φ*_*N*_〉 and 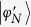 are expected ones.

Let us consider another set of conditional probabilites *p*_*mn*_ = *δ*_*mn*_ and *p*_*iN*_ = *p*_*Nj*_ = *p*_*NN*_ = 1/*N*. This corresponds to a case when Eve performs measurement in the encoding bases on each quantum states received, and announces 1 (0) when the same (different) outcomes are obtained. Analogously we get 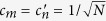. Then we obtain 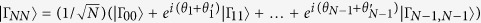. Combined by normalization condition, we get that all |Γ_*mm*_〉’s are orthogonal each other. The righthandside terms in Eqs (4) and (5) are *N* and there is no security clearly.

## Discussion and Conclusion

In principle, the method is applicable to other set of conditional probabilities physically realizable. Within Eq. (3) with given conditional probabilities, optimize the bounds in Eqs (4) and (5). However, it does not seem to be feasible because of its complexity. Robustness of the method can be shown by the fact that functions involved here are all continuous. If the set of conditional probabilities are arbitrarily close to the ones discussed above, the bounds are also arbitrarily close to the given ones. Only with the assumption about dimensionality, security was obtained. It seems to be worthwhile to search for application of the method in other tasks in quantum information processing.

Good candidates for real implementation of the *N*-dimensional states seems to be time-bins of single photons which are adapted in the phase-reference-free MDI QKD^15^,^19^ and round-robin-differential-phase-shift QKD^20^,^21^,^22^. Here the checking state can be made by opening optical switches such that all time-bins have non-zero possibility to contain a photon. Also spatial-bins may be a good candidate^23^.

In conclusion, we studied *N*-dimensional MDI QKD where one checking state is used. With the assumption about dimensionality, Eq. (1), we showed that the protocol is secure in zero QBER case. In the case when Eve’s does full measurement attack, the method also works. This suggests possibility of the protocol.

## Additional Information

**How to cite this article**: Hwang, W.-Y. *et al. N*-dimensional measurement-device-independent quantum key distribution with *N*+1 un-characterized sources: zero quantum-bit-error-rate case. *Sci. Rep.* **6**, 30036; doi: 10.1038/srep30036 (2016).
